# Urinary stone composition analysis and clinical characterization of 1520 patients in central China

**DOI:** 10.1038/s41598-021-85723-3

**Published:** 2021-03-19

**Authors:** Daling Zhang, Songchao Li, Zhengguo Zhang, Ningyang Li, Xiang Yuan, Zhankui Jia, Jinjian Yang

**Affiliations:** 1 Department of Urology, The First Affiliated Hospital of Zhengzhou University, No. 1 Jianshe East Rd., Zhengzhou, 450052 People’s Republic of China; 2 Urological Institute of Henan, Zhengzhou, 450052 Henan Province People’s Republic of China

**Keywords:** Risk factors, Urology

## Abstract

A total of 1520 patients with urinary stones from central China were collected and analysed by Fourier transform infrared spectroscopy between October 1, 2016 and December 31, 2019. For all patients, age, sex, comorbidities, stone location, laboratory examination and geographic region were collected. The most common stone component was calcium oxalate (77.5%), followed by calcium phosphate (8.7%), infection stone (7.6%), uric acid (UA) stone (5.3%)and cystine (0.9%). The males had more calcium oxalate stones (p < 0.001), while infection stone and cystine stones occurred more frequently in females (p < 0.001). The prevalence peak occurred at 41–60 years in both men and women. UA stones occurred frequently in patients with lower urinary pH (p < 0.001), while neutral urine or alkaline urine (p < 0.001) and urinary infection (p < 0.001) were more likely to be associated with infection stone stones. Patients with high levels of serum creatinine were more likely to develop UA stones (p < 0.001). The proportion of UA stones in diabetics was higher (p < 0.001), and the incidence of hypertension was higher in patients with UA stones (p < 0.001). Compared to the other types, more calcium oxalate stones were detected in the kidneys and ureters (p < 0.001), whereas struvite stones were more frequently observed in the lower urinary tract (p = 0.001). There was no significant difference in stone composition across the Qinling-Huaihe line in central China except UA stones, which were more frequently observed in patients south of the line (p < 0.001).

## Introduction

Urinary stones are a common disease whose prevalence is increasing worldwide, especially in developed countries. The prevalence of kidney stones in China is estimated to be approximately 5.8% according to the most recent epidemiological study performed in China^1^. Stone composition is affected by dietary habits, geography, socioeconomic conditions, infections, urinary tract anatomical deformity, and metabolic disorders^2^. Defining stone composition is important for determining a treatment plan, understanding aetiology and preventing recurrence. Fourier transform infrared (FT-IR) provides useful analysis of stone type and is applicable to smaller stone samples^3^; now, FT-IR has been widely used in clinical practice. Therefore, FT-IR analysis of stone composition is recommended during the evaluation of patients with urinary stones^4^.

To date, there are few studies of regional urinary stone composition in China^2,5–8^, which were conducted in economically prosperous regions in China. The central region of China, especially Henan province, is dominated by the agricultural population, with a long-term lag in economic development, and its climate, diet, and ethnicity are quite different from those of other regions. To date, no studies have been reported on the composition of calculi that occur in central China specifically. Our centre is one of the largest urinary stone management institutes in central China, and we performed a retrospective review of the stone compositions in central China, mainly Henan Province. The purpose of the present study is to determine whether differences in sex, age, geographic region and clinical characterizations may account for differences in stone composition.

## Materials and methods

The research proposal was approved by the First Affiliated Hospital of Zhengzhou University Research Ethics Committee (reference number 2020-KY-144).

Patients diagnosed with urolithiasis in The First Affiliated Hospital of Zhengzhou University from Oct 2016 to Dec 2019 were included. Only data from patients who experienced their first episode of urolithiasis and were underwent surgery were collected. Finally, 1520 stones were included. The stone fragments were collected intraoperatively, including during ureteroscopy, percutaneous nephrolithotomy (PCNL), cystolithotripsy and open surgery. All patients’ clinical data and demographic information, including age, sex, clinical biochemical indexes, stone location, and geographic region, were collected. Clinical biochemical indexes included routine urinalysis, urine culture and blood chemistry studies, which included the serum uric acid, creatinine, calcium, phosphorus, sodium and potassium levels. Blood and urine samples were taken from patients before breakfast. Routine urinalysis and urine culture were used to determine the occurrence of urinary tract infections. Urine is considered acidic when its PH ≤ 5.5, otherwise it will be considered neutral or alkaline. According to the long-term residence area, patients were divided into south of the Qinling Mountains-Huaihe River Line group (including Hubei Province, Hunan Province, Anhui Province, and the Nanyang, Zhumadian and Xinyang areas in Henan Province) and north of the Qinling Mountains-Huaihe River Line group (Shanxi Province, Hebei Province and other areas in Henan Province).

The stones were analysed by Fourier transform infrared spectroscopy. First, the stones were washed, dried at 75 °C and fully pulverized. Then, we mixed the stone powder (1 mg) with dry potassium bromide (200 mg); afterwards, the mixture was ground to the micrometre level in an agate pestle. The resulting mixture was compressed into a translucent sheet by exerting a standard pressure of 10 kPa. Finally, the sheet was analysed by Shimadzu FT-IR 8300 (Shimadzu Corporation, Japan). The resulting spectrum was then compared with all the reference spectrum for the known components of stones, allowing a precise analysis of the complex crystal mixture for each crystal type^9^. Stones were classified according to the Mayo Clinic stone classification practices and the guidelines of the European Urological Association^4,10^. Stones were classified as calcium oxalate (CaOx) if any kind of calcium oxalate (calcium oxalate monohydrate or calcium oxalate dihydrate) composed of > 50% of the stone. Stones were classified as calcium phosphate (CaP) stones if they contained a majority (50%) of carbapatite or if they contained any tricalcium phosphate, brushite, or amorphous calcium phosphate. Stones were classified as uric acid stones if they contained > 50% uric acid, uric acid dihydrate. Stones containing > 10% struvite, ammonium acid urate or monosodium urate monohydrate were categorized into the infection stone group. Similarly, stones containing any cystine were classified into the cystine group.

Differences in categorical variables between the groups were analysed using chi-squared tests. One-way ANOVA was used to compare the blood chemistry values among stone groups. SPSS statistical software 22 was used for analyses (SPSS, Inc., Chicago, IL, USA). A p-value < 0.05 was considered statistically significant.

### Ethics approval and consent to participate

The study received the approval from Ethics Committee of the First Affiliated Hospital of Zhengzhou University Research Ethics Committee (reference number 2020-KY-144). All procedures performed in studies involving human participants were in accordance with the ethical standards of the institutional research committee and with the 1964 Helsinki declaration and its later amendments or comparable ethical standards. This study is a retrospective study, and we applied to the First Affiliated Hospital of Zhengzhou University for exemption from informed consent. The exemption of informed consent is embodied in the ethical review certificate.

### Consent for publication

The authors agree for publication.

## Results

### Population characteristics

A total of 1520 stones were analysed in our study, 1036 (68.2%) of which were from males, with a male-to-female ratio of 2.14:1. Of the stones, 36 (2.3%) were collectedfrom children and adolescents 1–18 years old, 451 (29.7%) from patients 19 to 40 years old, 812 (53.4%) from patients 41 to 60 years old and 221 (14.5%) from patients 61 to 87 years old (Table 1). The average age of the patients was 46.3 ± 13.7 years, ranging from 1 to 87 years. Stones were found in the upper urinary tract in 1474 patients, with 923 (60.7%) in the kidney and 551 (36.3%) in the ureter, while stones were found in the lower urinary tract in 46 patients, with 42 (2.8%) in the bladder and 4 (0.2%) in the urethra (Table 1).

**Table 1:** Characteristics of urinary stones of according to gender of 1520 patients.

Characteristic	Overall	Male	Female
**Age (year)**			
Mean ± SD	46.88 ± 13.84	45.34 ± 13.76	48.25 ± 13.41
1–10	22	16	6
11–20	20	15	5
21–30	164	122	42
31–40	281	206	75
41–50	399	276	123
51–60	413	265	148
61–70	171	106	65
71–80	46	27	19
81–90	4	3	1
**Localization, n**			
Kidney	923	605	318
Ureter	551	396	155
Bladder	42	32	10
Urethra	4	3	1
**Region, n**			
North of the line^a^	1151	801	350
South of the line^a^	369	235	134
**Urinary infection, n**			
Yes	348	200	148
No	1172	836	336

^a^The Qinling Mountain-Huaihe River Line.

### Stone composition

Of 1520 stones, only 274 (18.0%) stones had one component, 1024 (67.4%) stones consisted of two components, and 222 (14.6%) stones consisted of three or more components (Table 2). It is worth noting that all struvite stones are mixed stones. We detected calcium oxalate in 1352 (88.9%) stones, and calcium oxalate accounted for more than 50% of the stone composition in 1178 (77.5%) stones. Similarly, calcium phosphate was found in 525 (34.5%) stones, and 132 (8.7%) stones contained more than 50% calcium phosphate. The remaining stone compositions included infection stone, 115 (7.6%); UA, 81(5.3%); and cystine, 14 (0.9%) (Table 3).

**Table 2:** The distribution of stones composed of single or multiple components.

Stones with single component	n	Stones with two components	n	Stones with three or more components	n
COM	174	COM/COD	701	COM/COD/carbapatite	69
anhydrous uric acid	50	COM/carbapatite	156	carbapatite/COM/COD	34
Carbapatite	18	Carbapatite/COM	36	Struvite/carbapatite/hydroxyapatite	29
COD	12	Struvite/carbapatite	34	Struvite/carbapatite/COM	26
Cystine	11	COD/carbapatite	32	Struvite/carbapatite/COM	21
Amorphous calcium phosphate	5	Carbapatite/COD	21	COD/COM/carbapatite	14
Brushite	2	Anhydrous uric acid/COM	19	COM/carbapatite/COD	12
Ammonium acid urate	2	COD/COM	13	Carbapatite/COM/anhydrous uric acid	5
		struvite/amorphous calcium phosphate	8	carbapatite/COM /anhydrous uric acid	4
		Struvite/COM	1	Struvite/COM/carbapatite	3
		Carbapatite/hydroxyapatite	1	COM/carbapatite/hydroxyapatite	3
		Cystine/COM	1	Struvite/ammonium acid urate/carbapatite	2
		Cystine/amorphous calcium phosphate	1		
Overall	274		1024		222

*COM* calcium oxalate monohydrate, *COD* calcium oxalate dihydrate.

**Table 3:** The distribution of main urinary stone composition.

Stone Compositions	Number of patients (%)
Calcium oxalate	1178(77.50)
Calcium oxalate-monohydrate	1121
Calcium oxalate-dihydrate	57
Calcium phosphate	132(8.68)
Carbapatite	125
Amorphous calcium phosphate	5
Brushite	2
Infection stone	115(75.7)
Struvite	113
Monosodium urate monohydrate	1
Ammonium acid urate	1
Urate stone	81(5.33)
Anhydrous uric acid	79
Uric acid dihydrate	2
Cystine	14(0.92)
Overall	1520

### Stone composition by sex and age

**Figure 1:**
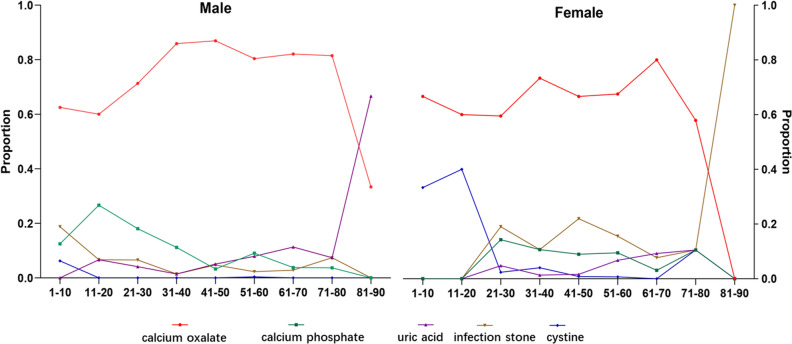
Proportions of stone compositions in different genders and age groups.

The most common type among male patients was calcium oxalate stones (81.7%), followed by calcium phosphate (8.6%), UA (5.7%), infection stone (3.9%), and cystine stones (0.2%). Calcium oxalate stones (68.6%) were the most common stones in females, followed by calcium phosphate (8.9%), infection stone (15.5%), UA (4.5%), and cystine stones (2.5%). Significant differences were observed between males and females in stone composition (Table 4). Calcium oxalate stones were preponderant in both sexes. Specifically, the proportions of calcium oxalate (p < 0.001) were much higher in males than in females, while the proportions of infection stone (p < 0.001) and cystine stone (p < 0.001) were much higher in women, as shown by a sex ratio of 0.625 and 0.17, respectively, compared with the overall male to female sex ratio of 2.19. To investigate the effects of age on stone composition, patients were divided into 1- to 10-year-olds, 11- to 20-year-olds, 21- to 30-year-olds, 31- to 40-year-olds, 41- to 50-year-olds, 51- to 60-year-olds, 61- to 70-year-olds, 71- to 80-year-olds and 81- to 90-year-olds. The highest stone prevalence appeared in 41- to 60-year-olds for both sexes, and the lowest incidence for both males and females was in 1- to 20-year-olds. The largest ratio of males to females was also observed in adolescence, and the ratio gradually decreased with age, from 2.89 in adolescents to 1.62 among patients older than 60 years old. The proportion of different stone types varied by sex and age (Fig. 1). The proportion of UA stone seemed to increase with age, accounting for 31.3% of stones in patients older than 60 years.

**Table 4:** Characteristics of patients stratified by stone composition.

Characterization	n	Compositions						
		CaOx	CaP	Struvite	UA	Cystine	χ^2^	P
**Gender**							85.9	< 0.001
Male	1036	846	89	40	59	2		
Female	484	332	43	75	22	12		
**Age**							112.3	< 0.001
1–18	35	22	3	4	1	5		
19–40	452	348	62	27	11	4		
41–60	813	635	58	68	47	5		
≥ 60	220	173	9	16	22	0		
**Localizations**							43.3	< 0.001
Kidney	923	702	75	80	55	11		
Ureter	551	453	50	26	21	1		
Bladder	42	21	6	8	5	2		
Urethra	4	2	1	1	0	0		
**Urinary infection**							88.6	< 0.001
Yes	348	220	36	65	23	4		
No	1172	958	96	50	58	10		
**Urine pH**^**a**^							32.8	< 0.001
Acidity	535	423	42	19	43	8		
Neutral or alkaline	985	755	90	96	38	6		
**Hypertension**							28.8	< 0.001
Yes	373	301	23	92	39	5		
No	1060	877	109	23	42	9		
**Diabetes**							28.2	< 0.001
Yes	158	105	19	10	21	3		
No	1362	1073	113	105	60	11		
**Line**^**b**^							22.3	< 0.001
North	1151	902	108	84	45	12		
South	369	276	24	31	36	2		

*CaOx* calcium oxalate, *CaP* calcium phosphate, *UA* urate stone.

^a^Urine is considered acidic when its PH ≤ 5.5, otherwise it will be considered neutral or alkaline.

^b^The Qinling Mountain-Huaihe River Line.

### Stone composition by clinical features and region

Calcium oxalate stones were more likely to be detected in patients with upper urinary tract stones (p < 0.001), while infection stones were more likely to be detected in patients with lower urinary tract stones (p = 0.001) (Table 4). Compared with patients with other types of stones, the patients with UA stones had higher mean values of serum uric acid and creatinine (p < 0.001). All other serum variables shown in Table 5, including calcium, phosphorus, sodium, and potassium, were not significantly different. The prevalence of UA stones in diabetic patients was higher than that in nondiabetic patients (p < 0.001). The incidence of hypertension was higher in patients with UA stones (p < 0.001). Patients with urinary infection had more infection stones (p < 0.001), while patients without urinary tract infection had more calcium oxalate stones. Lower urinary pH (≤ 5.5) was associated with UA stones (p < 0.001), while neutral urine or alkaline urine was more associated with struvite stones (p < 0.001). Moreover, infection stone was also closely associated with urinary tract infection (p < 0.001). The proportion of UA stones south of the Qinling Mountains-Huaihe River Line was higher than that north of the line (p < 0.001). The other stone compositions were not significantly associated with geographical region across the line.

**Table 5:** Serum biochemical values of all type stones forming individuals.

Serum biochemical	Total Sample	Compositions						P
		CaOx	CaP	Infection stone		UA	Cystine	
Calcium (mmol/L)	2.28 ± 1.34	2.28 ± 0.13	2.30 ± 0.14	2.30 ± 0.15		2.28 ± 0.13	2.28 ± 0.18	0.13
Potassium (mmol/L)	4.30 ± 0.46	4.30 ± 0.46	4.34 ± 0.49	4.33 ± 0.42		4.20 ± 0.44	4.55 ± 0.40	0.07
Phosphorus (mmol/L)	1.15 ± 0.21	1.15 ± 0.21	1.15 ± 0.20	1.19 ± 0.21		1.16 ± 0.24	1.29 ± 0.26	0.06
Magnesium (mmol/L)	0.98 ± 0.11	0.98 ± 0.11	0.97 ± 0.13	1.00 ± 0.09		0.96 ± 0.13	0.93 ± 0.10	0.05
Urate (mmol/L)	323.39 ± 94.71	320.41 ± 89.78	331.64 ± 86.49	291.71 ± 134.39		378.07 ± 80.93	440.07 ± 175.37	0.001
Creatinine (umol/L)	90.65 ± 66.95	85.72 ± 36.92	92.73 ± 46.51	77.39 ± 37.90		178.44 ± 225.71	87.43 ± 24.76	0.001

*CaOx* calcium oxalate, *CaP* calcium phosphate, *UA* uric acid.

## Discussion

China, especially the southern part, is in an area with a high incidence of stone disease. FT-IR helps to determine stone composition, further identify the aetiology of urolithiasis, and provide individualized treatment to accurately prevent stone recurrence^11^. Our study reveals that the urinary stone composition in central China has its own characteristics compared with those in other areas in China, which is highly correlated with sex, age, stone location, clinical biochemical indexes and geographic location.

In our study, the prevalence of CaOx (78.37% of stones) was higher than that in other regions in China^2,5,8,12,13^. The mainly vegetarian diet in central China may lead to this high CaOx prevalence. Several studies have demonstrated that dietary oxalate notably leads to urinary oxalate excretion^14,15^. The amount of urinary oxalate excretion is a significant factor in the development of CaOx stones. Patients with calcium oxalate stones benefit from decreased oxalates in their daily diet^16,17^.

UA stones are second only to calcium-containing stones in prevalence. In our research, UA stones were observed in 5.3% of cases, which was apparently lower than previous data in nationwide urinary stone composition analysis that found that urate stones (exactly speaking,mainly UA stones) comprised approximately 12.4% of cases^2^. The present study emphasizes that the factors that drive UA stone formation seem to be age and urinary pH. Khashayar sakhaee et al.^18^ found that a defect in ammonium excretion would account for the undue urinary acidity; then, increased urine acidity promotes uric acid supersaturation. Urinary pH is the crucial determinant of uric acid crystallization^19^. The proportion of UA stones consecutively grew with age, accounting for 2.9% in the age group of 1–18 years to 10.9% in patients aged over 60 years. Moreover, our study confirmed findings in previous studies that the mean age was higher in UA stone formers than in the other four age groups. Several studies have shown a similar increased proportion of UA stones with ageing. The findings relate to changes in kidney function with age. In this study, UA stones were associated with higher levels of serum Cr and uric acid, which seems to indicate a relationship between UA stones and mild renal injury. There was only weak evidence of the association between UA stone formation and hypercreatininaemia and hyperuricaemia. Furthermore, ageing, diabetes, insulin resistance and obesity are related to lower urinary pH and UA stones^20^.

Although revealing almost identical proportions of calcium-containing calculi, the figure in our study was considerably higher than that in previous reports in eastern China that found struvite stones comprised approximately 0.60–1.68% of urinary stones^5,21^. The proportion of infection stones detected in our study was 7.6%, which was higher than the national average^2^. This higher incidence of infection stones might result from patients coming from central China, especially in the vast rural areas of Henan Province, with a lower socioeconomic status and standard of medical care. Knoll et al.^22^ hypothesized that the higher prevalence of infectious stones might result from poor medical care in eastern Germany. As a regional centre for stone treatment, our centre dealt with more complex cases of staghorn calculi and urinary calculi with severe infections, which could lead to a selection bias. Consistent with the results in other reports^2,5,7,8,10,12^, we found that the proportion of infection stones among females in the present study was three times that in males, accounting for 15.5% and 3.9%, respectively. Another finding of our research was that the rate of infection stone is apparently related to urinary tract infection and a higher urine pH profile. Persistent urinary tract infection with urease-producing bacteria will increase the pH of the urine and thus facilitate the formation of infection stone^23^. All of the struvite stones in our study were multicomponent, which shows that the mechanism of struvite formation is complex and that the specific formation process needs further study.

Many studies about urolithiasis have revealed a male predominance of the disease, with the ratio varying from 1.1:1 to 7.6:1^8,10,24^. Our study came to the same conclusion, while it is worth noting that the proportion of males and females with urinary calculi decreases with age. Although our data are not representative of epidemiological statistics, the reasons behind this result are still worth investigating. Prior studies have shown that postmenopausal women are more likely to develop kidney stones^25^. Oestrogen status might be one of the reasons for the sex differences in stone incidence.

In our study, kidney stones and ureteral stones were more commonly identified as calcium oxalate stones, and bladder stones and urethra stones were more generally confirmed as infection stones, which indirectly suggested that the causes of upper and lower urinary tract stones are different. Previous studies have shown that the severity of diabetes is significantly related to the risk of kidney stones^26–28^. However, in those studies, the patient's stone history was obtained by questionnaire survey, and stone composition was not included, so the effect of diabetes on the composition of the stones could not be assessed. Our study revealed that diabetes patients had a greater likelihood of developing UA stones. Insulin resistance, which is a classic trait of diabetes mellitus, may increase the risk of UA stone formation^29^.

Traditionally, the Qinling Mountain-Huaihe River Line geographically divides China into south and north, which makes obvious differences in climate, water system, vegetation, diet, etc. However, it is worth noting that we found no difference in the stone composition across the line in central China except for UA stones. South of the line, the high proportion of urate stones may be related to a hot and dry climate and the local high-protein diet. Compared with the nationwide analysis of stone composition^2^, the distribution of patients in our study was relatively concentrated, so it is no surprise that there is no significant difference in stone composition in central China.

Our study has limitations. First, all stones were collected during surgery, which leads to a selection bias because of the exemption of asymptomatic stones or patients who passed stones naturally through the urethra. Second, as a single-center study from the specialty surgical center, our data may not reflect the true composition of stones in the population. Future efforts could be made to collect stone analyses information from other centers within our region. Third, all patients’ urine samples were taken in the morning, which may cause partial urine acidity. Finally, clinical data, such as 24-h urine analysis and body mass index, were not investigated.

## Conclusions

In summary, this study presented a regional stone composition analysis in a large population in central China. Our study presented a higher proportion of struvite stones in central China. Age, sex, stone location, comorbidities, and partial clinical biochemical indexes such as serum uric acid and creatinine values have a significant influence on stone composition. A more detailed and in-depth understanding of the composition of urolithiasis in specific populations will help to assess, treat and prevent the disease more effectively.

## Data Availability

The datasets used during the current study are available from the corresponding author or first author on reasonable request.

## Abbreviations

FTIR: Fourier-transform infrared
CaOx: Calcium oxalate
COM: CaOx monohydrate
COD: CaOx dihydrate
UA: Uric acid
PCNL: Percutaneous nephrolithotomy
Cr: Creatinine

## References

[1] Zeng G, Mai Z, Xia S (2017). Prevalence of kidney stones in China: An ultrasonography based cross-sectional study. BJU Int..

[2] Ye Z, Zeng G, Huan Y (2019). The status and characteristics of urinary stone composition in China. BJU Int..

[3] Khan AH, Imran S, Talati J (2018). Fourier transform infrared spectroscopy for analysis of kidney stones. Investig. Clin. Urol..

[4] Skolarikos A, Straub M, Knoll T (2015). Metabolic evaluation and recurrence prevention for urinary stone patients: EAU guidelines. Eur. Urol..

[5] Sun X, Shen L, Cong X (2011). Infrared spectroscopic analysis of 5248 urinary stones from Chinese patients presenting with the first stone episode. Urol. Res..

[6] Wu W, Yang D, Tiselius HG (2014). The characteristics of the stone and urine composition in Chinese stone formers: Primary report of a single-center results. Urology.

[7] Yang X, Zhang C, Qi S (2016). Multivariate analyses of urinary calculi composition: A 13-year single-center study. J. Clin. Lab. Anal..

[8] Wang S, Zhang Y, Zhang X (2020). Upper urinary tract stone compositions: The role of age and gender. Int. Braz. J. Urol..

[9] Álvarez JLG, Martínez MJT, Fernández MA (2012). Development of a method for the quantitative analysis of urinary stones, formed by a mixture of two components, using infrared spectroscopy. Clin. Biochem..

[10] Lieske JC, Rule AD, Krambeck AE (2014). Stone composition as a function of age and sex. Clin. J. Am. Soc. Nephrol..

[11] Moe OW (2006). Kidney stones: Pathophysiology and medical management. Lancet.

[12] Wu W, Yang B, Ou L (2014). Urinary stone analysis on 12,846 patients: A report from a single center in China. Urolithiasis.

[13] Ma RH, Luo XB, Li Q (2018). Systemic analysis of urinary stones from the Northern, Eastern, Central, Southern and Southwest China by a multi-center study. BMC Urol..

[14] Holmes RP, Goodman HO, Assimos DG (2001). Contribution of dietary oxalate to urinary oxalate excretion. Kidney Int..

[15] Holmes RP, Ambrosius WT, Assimos DG (2005). Dietary oxalate loads and renal oxalate handling. J. Urol..

[16] Noori N, Honarkar E, Goldfarb DS (2014). Urinary lithogenic risk profile in recurrent stone formers with hyperoxaluria: A randomized controlled trial comparing DASH (dietary approaches to stop hypertension)-style and low-oxalate diets—sciencedirect. Am. J. Kidney Dis..

[17] Shah S, Calle JC (2016). Dietary and medical management of recurrent nephrolithiasis. Cleve Clin. J. Med..

[18] Sakhaee K, Adams-Huet B, Moe OW (2002). Pathophysiologic basis for normouricosuric uric acid nephrolithiasis. Kidney Int..

[19] Moe OW, Abate N, Sakhaee K (2002). Pathophysiology of uric acid nephrolithiasis. Endocrinol. Metab. Clin. N. Am..

[20] Abate N, Chandalia M, Cabo-Chan AV (2004). The metabolic syndrome and uric acid nephrolithiasis: Novel features of renal manifestation of insulin resistance. Kidney Int..

[21] Jing Z, GuoZeng W, Ning J (2010). Analysis of urinary calculi composition by infrared spectroscopy: A prospective study of 625 patients in eastern China. Urol. Res..

[22] Knoll T, Schubert AB, Fahlenkamp D (2011). Urolithiasis through the ages: Data on more than 200,000 urinary stone analyses. J. Urol..

[23] Das P, Gupta G, Velu V (2017). Formation of struvite urinary stones and approaches towards the inhibition—A review. Biomed. Pharmacother..

[24] Daudon M, Doré JC, Jungers P, Lacour B (2004). Changes in stone composition according to age and gender of patients: A multivariate epidemiological approach. J. Urol. Res..

[25] Maalouf NM, Sato AH, Welch BJ (2010). Postmenopausal hormone use and the risk of nephrolithiasis: Results from the Women's Health Initiative hormone therapy trials. Arch. Intern. Med..

[26] Taylor EN, Stampfer MJ, Curhan GC (2005). Diabetes mellitus and the risk of nephrolithiasis. Kidney Int..

[27] Meydan N, Barutca S, Caliskan S (2003). Urinary stone disease in diabetes mellitus. Scand. J. Urol. Nephrol..

[28] Weinberg AE, Patel CJ, Chertow GM (2014). Diabetic severity and risk of kidney stone disease. Eur. Urol..

[29] Daudon M (2006). Type 2 diabetes increases the risk for uric acid stones. Am. Soc. Nephrol..

